# Antifungal activity of *Cleome gynandra* L. aerial parts for topical treatment of *Tinea capitis*: an in vitro evaluation

**DOI:** 10.1186/s12906-016-1187-9

**Published:** 2016-07-08

**Authors:** Lawrence Imanirampa, Paul E. Alele

**Affiliations:** Department of Pharmacy, Faculty of Medicine, Mbarara University of Science and Technology, P.O. Box 1410, Mbarara, Uganda; Department of Pharmacology and Therapeutics, Faculty of Medicine, Mbarara University of Science and Technology, P.O. Box 1410, Mbarara, Uganda

**Keywords:** Aerial parts, Antifungal, *Cleome gynandra*, in vitro, *Tinea capitis*

## Abstract

**Background:**

*Cleome gynandra* L. (Capparaceae) is an edible weed used in Uganda topically for its presumed antifungal activity against *Tinea capitis.* The goal of this study was to determine if this plant possesses antifungal activity in vitro, since *T. capitis* is a pervasive infection among especially rural children.

**Methods:**

Antifungal activity assay was performed by Broth dilution method, and testing done on clinical isolates of three common *Tinea capitis*-causing fungal strains. Evaluation of in vitro antifungal activity of the ethanol and water extracts of *C. gynandra* was done to determine the minimum inhibitory concentrations (MICs) and the minimum fungicidal concentrations (MFCs) of the extracts.

**Results:**

The MIC of *C. gynandra* ethanol extract ranged from 0.0313 to 0.0625 mg/ml for *Trichophyton rubrum,* and from 0.25 to 0.5 mg/ml for both *Microsporum canis* and *Trichophyton mentagrophytes*. The MICs of *C. gynandra* aqueous extract ranged between 0.125 to 0.25 mg/ml for *T. rubrum,* and 0.25 to 0.5 mg/ml for both *M. canis* and *T. mentagrophytes. T. rubrum* was more sensitive than *M. canis* (*p* < 0.002) and more sensitive than *T. mentagrophytes* (*p* < 0.035) to the antifungal activity of *C. gynandra. T. rubrum* was 6.9 times (95 % CL: 1.15 – 41.6) more likely to have a better outcome (more sensitive) than *T. mentagrophytes. Cleome gynandra* aqueous extract had MFC of ≥0.0313 mg/ml for *M. canis,* ≥0.0156 mg/ml for *T. mentagropyhtes,* and ≥0.0625 mg/ml for *T. rubrum. Cleome gynandra* ethanol extract showed MFCs of ≥0.5 mg/ml for *M. canis* and *T. mentagrophytes,* and *≥*0.125 mg/ml for *T. rubrum.*

**Conclusion:**

Both plant extracts demonstrated antifungal activity, shown by the MIC and MFC for the different extracts, which varied with the type of organism of the clinical fungal isolates. The ethanol extract exhibited comparable antifungal activity to the aqueous extract indicated by the MIC values seen. Conversely, after subculturing the fungal isolates, MFCs were lower for the aqueous than for the ethanol extract.

## Background

Many herbal products are presumed to have antifungal properties but few scientific studies have been done to validate these claims [1]. *Cleome gynandra* is one such plant assumed to cure *Tinea capitis. Cleome gynandra L.* (*Isogi* in the Rufumbira language, Uganda; *Eshogi* in the Runyankole/Rukiga language, Uganda, and *Akeo* in Lango, Uganda), also known as African Spider flower, African Cabbage, and Dog Mustard, is an angiosperm that belongs to the family Capparaceae. Shoots and leaves of *C. gynandra* are crushed and used topically in southwestern and northern Uganda for management of *Tinea captitis* [2, 3] and also eaten as a vegetable food [3–5]. Other medicinal uses of *C. gynandra* include the treatment of migraine headache and epilepsy [6]; stomach ache [3]; ear pain and sepsis, diphtheria, vomiting, promotion of labor at the end of pregnancy, [7]; and snake bite [8]. The mature plant is a herb that grows to about 1.3 m with leaves that are compound (palmitate leaves) with five leaflets (why it is called African Spider plant). Its flowers are hermaphrodite, having both male (androceum) and female (gynandroceum) organs thus acquiring the name *Gynandropsis* – gynophore (female) and androphore (male). Cleome gynandra has thus also been called *Gynandropsis gynandra* L. Its flowers are regular (actinomophic), sometimes zygomorphic, hypogynous or perigynamous, and bisexual [9].

*Tinea capitis* is one of the most pervasive fungal infections in the population, mainly among children of school age below 12 years, and is prevalent in adults whose immunity has been suppressed [10]. Current treatment options include both topical and oral agents including clotrimazole, miconazole, nystatin, griseofluvin, and terbinafine. These drugs are expensive for most of the rural poor and have many side effects, yet many plants with medicinal activity probably exist. Many plants contain chemicals of medicinal value (secondary metabolites such as alkaloids, natural phenols, terpenoids, flavonoids and antibiotics) that are precursors of medicines. The largest rural populations in areas of developing countries [11, 12] use these plants, or their parts, for their presumed antifungal, antibacterial, antiviral, anthelmintic, and antimalarial activities. Some medicinal plants are known to treat fungal infections for example *Candida albicans;* Black walnut and Chamomile have been used to treat *C. albicans*. The tea tree has been used to treat athlete’s foot [13].

A number of plants are accordingly currently used in traditional medicine to treat diverse fungal infections. Some plants have been identified for the treatment of opportunistic infections, including oral fungal infections in HIV patients [6, 14]. Studies done in Tanzania on some Combretaceae and other families of plants indicate the possibility of obtaining plants with clinically useful antifungal activity, particularly against *C. albicans* and other yeast-like fungi [15]. Other plants studied in East Africa have been shown to have antifungal activity of varying amounts [6, 16]. These herbal treatments applied by traditional healers and rural populations need to be documented scientifically for their safety and efficacy, and feedback given to the community. Therefore, the present study was conducted to determine the in vitro antifungal activity of *Cleome gynandra*, a plant locally used for the treatment of *Tinea capitis* in children, given that fungal infections of the scalp are especially pervasive in children.

## Methods

### Study design

This was a controlled experimental in vitro study carried out at both the Pharmacy Laboratory and the Mycology Unit of the Microbiology laboratory at Mbarara University of Science and Technology. Three clinical fungal isolates, namely, *Microsporum canis*, *Trichophyton rubrum* and *Trichophyton mentagrophytes* were obtained from the Mycology Department of Mbarara Regional Referral Hospital. The fungal isolates had not lasted more than 3 months in the laboratory.

### Identification, collection and drying of *Cleome gynandra*

Fresh aerial plant parts (shoots, leaves, flowers and fresh pods) believed to have antifungal activity against *Tinea capitis* were collected from cultivated lands around Mbarara University of Science and Technology (MUST) and taken for botanical identification at the Faculty of Science, MUST. The plant was identified by Dr. Eunice A. Olet, a taxonomist at the Department of Biological Sciences, Faculty of Science, MUST, and given a herbarium number, IMANIRAMPA001. A herbarium specimen was prepared and deposited at the Department of Biological Sciences. The collected and identified plant materials were brought to the laboratory, washed with fresh distilled water, cut into small pieces, and air-dried under shade at room temperature. The dried plant materials were crushed into small pieces and blended using a portable electric blender to reduce them into powder form, and weight of the powder taken.

### Extraction

Extraction was done with ethanol using the Soxhlet extractor, and by cold maceration with fresh distilled water.

### Soxhlet extraction

A predetermined amount of the dried plant powder was put in a Soxhlet apparatus to which enough ethanol (70 % v/v) was added to submerge the powder and continuously extract it, until the extracting solvent became clear in the thimble indicating that the extraction of the phytochemicals soluble in the solvent ethanol was complete. The volume was noted; the extract was dried in an oven at a temperature of 70 °C centigrade, and the weight of the dry ethanol extract taken. The residue after the ethanol extraction process was dried for further extraction by cold maceration using fresh, cool, distilled water.

### Cold maceration

Extraction of the plant material was done using maceration as the appropriate method because when used as a phytomedicine, *Cleome gynandra* is applied to the affected scalp in the raw form, since it is believed that heating may inactivate the ingredients. The dried herbal material (after ethanol extraction) was recrushed into powder by use of a portable electric blender, its weight taken, then transferred into a glass vessel to which enough volume of the extracting solvent water was added until the medicinal plant residues were fully immersed. The vessel was closed with a tight-fitting glass cover and the contents in the vessel were shaken after every 4 h except at night, and left to stand for three days (72 h) but with subsequent agitation until this period was over. The contents of the flask were then strained through two clean pieces of cotton cloth placed on top of filter paper, both supported by a funnel, and the extracted solution (miscella) collected in a flask with a tight-fitting cover. The maximum yield of water extract was obtained by squeezing the marc (solid residue) in the top clean dry piece of cloth, while drippings of liquid extract were allowed to pass through the second clean dry piece of cloth, to the contents of the flask through Whatman’s filter paper. The volume of the yield was noted. Both the ethanol and water extracts were transferred to a hot-air oven for drying at temperatures between 50 °C and 70 °C, and later transferred into a desiccator for further drying.

### Phytochemical screening

Phytochemical screening of the extracts for secondary metabolites was done according to standard methods [11]. Briefly, general chemical composition of the plant was determined by means of chemical qualitative analysis on the aerial plant extracts obtained through extraction with solvents of increasing polarity, that is, ethanol (70 % v/v), and water, respectively. The phytochemical screening for secondary metabolites was carried out at the Pharmacy laboratory of MUST. Oils and fats were tested by adding one ml of Sudan III diluted with equal volume of water, adding a small quantity of each extract, and heating the mixture on a water bath until the solvent evaporated; a red color was observed for fat globules present in the mixture. Alkaloids were tested by adding a small quantity of each extract (ethanolic and aqueous) to a test tube and stirring with 5 mL of 1 % hydrochloric acid for five minutes on a water bath, and then filtering (Dragendorff’s test). To the filtrate of each extract, a few drops of Dragendorff’s reagent were added and then observed for the formation of a reddish-brown precipitate. Steroids were tested by dissolving small amounts of each extract in 1 ml of chloroform; 2 ml of acetic anhydride were added and mixed thoroughly, then a few drops of concentrated sulphuric acid were added from the sides of the test tube without shaking, and the solution observed for the formation of a brown ring at the junction of the two layers (Liebermann-Burchard’s test). Terpenoids were tested as follows: to the portion of each extract, two ml of chloroform was added and then a few drops of concentrated sulphuric acid added from the sides of the test tube without shaking and the solution observed for the formation of a deep red color indicating the presence of terpenoids (Salkowski’s test). Sugars were tested using Fehling’s test: a small quantity of each extract was hydrolyzed by boiling with 5 ml of dilute hydrochloric acid and the resulting solution neutralized with sodium hydroxide solution. To each of these solutions, a few drops of Fehling’s solution were added, each heated on a water bath for 2 min, and observed for the formation of a brick-red precipitate. Tannins were tested using the ferric chloride test. Small quantities of each extract were boiled in 10 ml of distilled water and then filtered. Few drops of 1 % ferric chloride solution were added to 2 ml of each filtrate and observed for green color changes. Lastly, flavonoids were tested using the lead acetate test in which a small quantity of each extract was added to 2 ml of lead acetate. A positive test was indicated by the formation of a precipitate.

### Antifungal bioassay

#### Selection of fungal strains

Fungal strains selected for the antifungal bioassays were clinical isolates of *Microporum canis, Trichophyton rubrum,* and *Trichophyton mentagrophytes* obtained from the Mycology Department of MUST as dermatophytic fungi causing *Tinea capitis.*

#### Test isolates

Clinical isolates of *M. canis, T. rubrum,* and *T. mentagrophyte* were identified using Lactophenol Cotton Blue Staining (LPCB) technique, based on their morphological structures including micro-conidia and macro-conidia. The pure vegetative colonies of test isolates were subcultured onto SDA slants incubated at 30 °C for 7 days and observed for growth. Two slants per fungal species were prepared. These slants were considered for the initial inocula. Using the tip of a sterile applicator stick, pure colonies of *M. canis, T. mentagrophyte* and *T. rubrum* each respectively, were picked and added to 5 mls of 0.85 % sodium chloride, and the resultant suspension was vortexed for 15–20 s and later adjusted to a 3.0 McFarland standard turbidity. Two hundred microliters (200 μl) of each fungal suspension were mixed separately with the plant extracts in their RPMI 1640 serial dilutions. Eight (8) tubes for each fungal suspension containing the plant extract dissolved in RPMI 1640 and chloramphenicol (40 μl), were arranged in descending order of their concentrations (0.5 mg/ml, 0.25 mg/ml, 0.125 mg/ml, 0.0625 mg/ml, 0.0313 mg/ml, 0.0156 mg/ml, 0.00781 mg/ml, and 0.00391 mg/ml). Two other tubes, one for positive control containing chloramphenicol, RPMI 1640, fluconazole and the fungi, and the other for negative control containing the fungal isolates, chloramphenicol and RPMI 1640, were included. Each of the three clinical fungal isolates was tested against *Cleome gynandra* ethanol and water extracts and against fluconazole. Three tests for ethanol and water extracts of different concentrations (0.5 mg/ml, 0.25 mg/ml, 0.125 mg/ml, 0.0625 mg/ml, 0.0313 mg/ml, 0.0156 mg/ml, 0.00781 mg/ml, and 0.00391 mg/ml) were performed to make thirty (30) tests and the same procedure was applied on the water extract. The overall number of tests carried out was sixty.

#### Dissolution of extracts

The dried ethanol and aqueous extracts of *C. gynandra* were weighed; 2.0 g of each extract was dissolved in 2 ml of RPMI and made to 1 g/ml. From these solutions, different strengths of starting concentrations (0.5 mg/ml, 0.25 mg/ml, 0.125 mg/ml, 0.0625 mg/ml, 0.0313 mg/ml, 0.0156 mg/ml, 0.00781 mg/ml, and 0.00391 mg/ml) were prepared by serial dilutions. These concentrations were used to test the antifungal activity of *C. gynandra* to obtain both the minimum inhibitory concentration (MIC) and the minimum fungicidal concentration (MFC).

#### Broth dilution method

Two hundred microliters (200 μl) of the confirmed fungal suspension were drawn each time, added to each of the test tubes as arranged serially, and mixed thoroughly. All test tubes contained chloramphenicol to kill bacterial contaminants. The positive control test tube had fluconazole (2 mg/ml) and the fungal isolates. Test tubes with their contents were transferred into the incubator to incubate between 25–30 °C for 24 h while observing for turbidity in each test tube, and each time comparing each test tube with the positive control and scoring the turbidity. The turbidity readings were scored numerically and using codes, for three days (72 h), to determine the MICs. Turbidity was scored and interpreted as follows [17]:

0 = optically clear/no visible growth, +1 = approximately 25 % growth control, +2 = approximately 50 % growth control, +3 = approximately 75 % growth control, +4 = no reduction in growth control.

The procedure was repeated for *T. mentagrophytes* and *T. rubrum.* On the third day a sample of the test isolates was picked with a cool heat-sterilized wire loop and subcultured onto SDA slant serially, incubated between 25–30 °C and observed for aberrant growth (appearance of filaments) for fourteen days to determine the MFCs.

#### Testing of *C. gynandra* extracts for antifungal activity on *Microsporum canis, Trichophyton mentagrophytes*, and *Trichophyton rubrum*

Each *C. gynandra* extract dilution was inoculated with 200 μl of fungal organisms and incubated at 25–30 °C for 24 h. The *Microsporum canis (Mc), Trichophyton mentagrophytes (Tm)* and *Trichophyton rubrum (Tr)* innocula were each picked separately using a cool heat-sterilized wire loop, cultivated in RPMI medium, and incubated between 25–30 °C for three days. MICs were determined by dermatophyte growth (turbidity) in each test tube while comparing with the positive control, and were scored numerically as follows 0 = optically clear/no visible growth, 1 = approximately 25 % growth control, 2 = approximately 50 % growth control, 3 = approximately 75 % growth control and 4 = very turbid (no reduction in growth control) according to CLSI, 2008 guidelines [17–19].

#### Endpoints for determining minimum inhibitory and minimum fungicidal concentrations

The MIC for dermatophyte was defined as the lowest drug concentration that inhibited visible growth of the fungi. The minimal fungicidal concentrations (MFCs) for dermatophytes were determined by subculturing fungal isolates from each test tube with no visible growth onto an SDA slant. The slants were incubated at 30 °C for 14 days while monitoring for fungal growth. The MFC was defined as the lowest concentration of drug that yielded a negative subculture [20].

#### Control tests

Two controls were used for each clinical fungal isolate. Six positive controls were used, each containing RPMI 1640, chloramphenicol, fluconazole and fungi, and six other negative controls were also used, each containing RPMI 1640, chloramphenicol, plus the fungal isolates, but without the plant extracts. For the positive controls, one ml of RPMI 1640, 40 μl chloramphenicol (2 g/10 ml), fluconazole (2 mg/ml), and 200 μl fungi were added into sterile tubes and mixed to obtain a uniform mixture. One ml of this mixture was then added onto the surface of a sterile SDA slant, spread over the medium, and observed for 14 days for filament growth. For the negative controls, 200 μl of clinical fungal isolates were added to 1 ml of sterile RPMI 1640 (suspension) to which an antibacterial agent (chloramphenicol) was added into a sterile test tube, uniformly mixed, and incubated at 30 °C for 72 h while observing for turbidity. Media controls were made by adding the mixture of RPMI (I ml), chloramphenicol (40 μl), and bacteria (staphylococcal species), incubating at 30 °C for 72 h in a sterile test tube; another ml of RPMI 1640 was spread on SDA slant and uninoculated SDA slant was also incubated at the same temperature (30 °C) for 72 h. Quality control strain of *Candida albicans* (ATCC 90028) was inoculated on SDA, incubated between 25–30 °C, and purity checked and confirmed from its structural morphology using a gram stain and germ tube. Quality control was performed using *C. albicans* every time a positive control was tested and the extracts and a set of clinical isolates were evaluated.

#### Reading of minimum inhibitory and minimum fungicidal concentrations

MICs of both plant ethanol and water extracts were determined visually from the first day the growth control (negative control) showed fungal growth/turbidity, and 72 h later. Growth/turbidity in each test tube was given a score or code by comparing its contents with those of the positive control (clear). The MFCs of both ethanol and water extracts were determined by picking 100 μl of dermatophyte from each test tube and subculturing this onto an SDA slant, incubating at 25–30 °C, and visually observing for filamentous growth on the SDA slants in all test tubes while comparing with the positive and negative controls. Growth on each SDA slant was coded as Fgo where filamentous growth was observed, or as Nfgo for no filamentous growth observed.

#### Data analysis

Because of the ordinal nature of the turbidity as an indicator of minimal inhibitory concentrations (expressed as level of turbidity), we used an ordinal logistic regression analysis based on fitting a proportional odds model. We analyzed the effects of the two independent variables (fungal strain and extract), on turbidity (minimum inhibitory concentrations). Odds ratios-pairwise comparisons of the categorical variables (fungal strain and ethanol extract) were also determined. Maximum likelihood estimates were obtained and shown as *p*-values. Statistical analysis was done using SAS® version 9 software.

#### Ethical considerations

The research proposal was reviewed and approved by the Faculty Research and Ethics Committee (FREC) and the Institutional Review Committee (IRC) of Mbarara University (approval numbers are 03/08-12, and MUIRC 1/7, respectively). The hospital administration of Mbarara Regional Referral Hospital approved the use of the human clinical fungal isolates, and laboratory rules and protocols during sensitivity testing were strictly followed.

## Results

Percentage yields of the dried extracts were calculated as weight of dried concentrate divided by the initial weight of powder ×100; the percentage yield was 23.4 % for the dry ethanol extract, and 13.3 % for the dry aqueous extract.

### Phytochemical screening for constituents of *Cleome gynandra* ethanol and water extracts

Phytochemical screening of *C. gynandra* aerial parts of both plant extracts indicated the presence of alkaloids, flavonoids, tannins, terpenoids, steroids; however, oils, fats, and steroids were absent in the water extract, but present in the ethanolic extract.

### Antifungal activity of *C. gynandra*

**Fig. 1:**
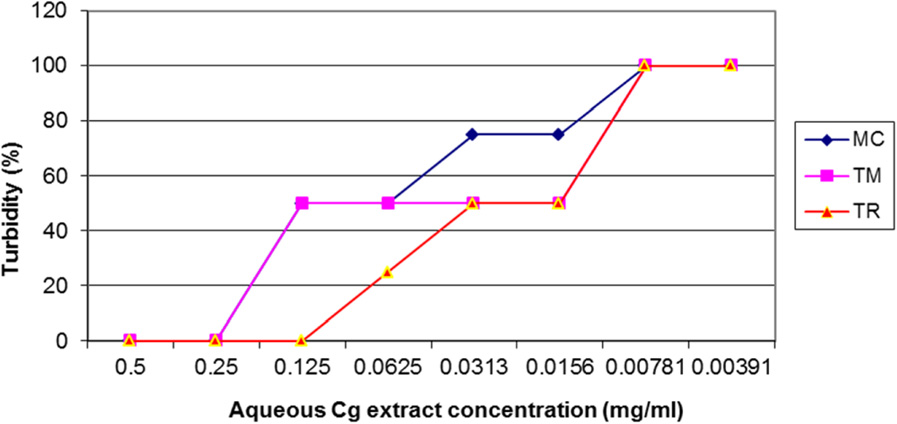
MICs of aqueous extract of *C. gynandra* against the fungal isolates. Minimum inhibitory concentrations (MICs) of aqueous extract of *Cleome gynandra* against the clinical fungal isolates ranged from 0.0625 to 0.125 mg/ml for *Trichophyton rubrum* and 0.125 to 0.25 mg/ml for both *Trichophyton mentagrophytes* and *Microsporum canis*. MC = *Microsporum canis*; TM = *Trichophyton mentagrophytes*; and, TR = *Trichophyton rubrum*

**Fig. 2:**
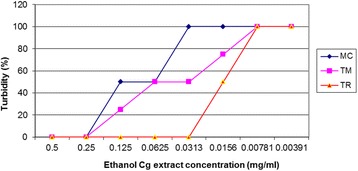
MICs of ethanol extract of *C. gynandra* against the fungal isolates. Minimum inhibitory concentrations (MICs) of ethanol extract of *Cleome gynandra* ranged from 0.125 to 0.25 mg/ml for both *Microsporum canis* and *Trichophyton mentagrophytes* and 0.0156 to 0.0313 mg/ml for *Trichophyton rubrum*; MC = *Microsporum canis*; TM = *Trichophyton mentagrophytes*; and, TR = *Trichophyton rubrum*

MIC readings were determined by reading or observing levels of turbidity by assigning numbers that were interpreted in percentages against the concentrations in milligrams/ml (mg/ml). The MICs of the aqueous extract of *C. gynandra* against the clinical fungal isolates ranged from 0.0625 to 0.125 mg/ml for T. rubrum and 0.125 to 0.25 mg/ml for both *T. mentagrophyte* and *M. canis* (Fig. 1). For the ethanol extract of *Cleome gynandra*, MICs ranged from 0.125 to 0.25 mg/ml for both *Microsporum canis* and *Trichophyton mentagrophytes*, and between 0.0156 to 0.0313 mg/ml for *Trichophyton rubrum* (Fig. 2).

The aqueous extract of *C. gynandra* had MFCs of  ≥ 0.0313 mg/ml for *M. canis,* ≥0.0156 mg/ml for *T. mentagropyhtes* and ≥ 0.0625 mg/ml for *T. rubrum* during the course of 14 days (Table 1). There was filamentous growth in the negative control (−ve ctrl) with a score of +4 corresponding to 100 %, and no filamentous growth in positive control (+ve ctrl) with score of zero (Table 1).

**Table 1:** MFCs of aqueous extract of *C. gynandra* against the fungal isolates

Fungi	Minimum fungicidal concentrations of aqueous extract of *Cleome gynandra* in mg/ml								Controls	
	0.5	0.25	0.125	0.0625	0.0313	0.0156	0.00781	0.00391	-ve ctrl	+ve ctrl
Mc	Nfgo	Nfgo	Nfgo	Nfgo	Nfgo	Fgo	Fgo	Fgo	Fgo	Nfgo
Tm	Nfgo	Nfgo	Nfgo	Nfgo	Nfgo	Nfgo	Fgo	Fgo	Fgo	Nfgo
Tr	Nfgo	Nfgo	Nfgo	Nfgo	Fgo	Fgo	Fgo	Fgo	Fgo	Nfgo

Fgo denotes that filamentous growth was observed, while Nfgo denotes that no filamentous growth was observed. The results obtained during the course of 14 days revealed that the water extract of *Cleome gynandra *had MFCs of ≥ 0.0313 mg/ml for *Microsporum canis*, ≥0.0156 mg/ml for *Trichophyton mentagrophytes* and ≥ 0.0625 mg/ml for *Trichophyton rubrum*. There was filamentous growth in the negative control (- ve ctrl) with a score of +4, corresponding to 100%, and no filamentous growth in positive control (+ve ctrl), with a score of zero (0). Mc = *Microsporum canis*; Tm = *Trichophyton mentagrophytes*; and, Tr = *Trichophyton rubrum*

Conversely, the MICs of ethanol extract of *C. gynandra* ranged from 0.125 to 0.25 mg/ml for both *M. canis* and *T. mentagrophytes* and 0.0156 to 0.0313 mg/ml for *T. rubrum* (Fig. 2). The MFCs of the ethanol extract of *C. g*ynandra was ≥ 0.5 mg/ml for *M. canis* and *T. mentagrophytes,* and ≥ 0.125 mg/ml for *T. rubrum* as a result of subculturing 200 μl of dermatophytes from each serially diluted test tube onto SDA slants (Table 2). The lowest average percentage turbidity was exhibited by the aqueous and ethanol extracts against *T. rubrum*, followed by *T. mentagrophyte* and finally, *M. canis* (Table 3).

**Table 2:** MFCs of ethanol extract of *C. gynandra* against the fungal isolates

Fungi	Minimum fungicidal concentrations of ethanol extract of *Cleome gynandra* in mg/ml								Controls	
	0.5	0.25	0.125	0.0625	0.0313	0.0156	0.00781	0.00391	−ve ctrl	+ ctrl
Mc	Nfgo	Fgo	Fgo	Fgo	Fgo	Fgo	Fgo	Fgo	Fgo	Nfgo
Tm	Nfgo	Fgo	Fgo	Fgo	Fgo	Fgo	Fgo	Fgo	Fgo	Nfgo
Tr	Nfgo	Nfgo	Nfgo	Fgo	Fgo	Fgo	Fgo	Fgo	Fgo	Nfgo

Fgo denotes that filamentous growth was observed, while Nfgo denotes that no filamentous growth was observed. The ethanol extract of *Cleome gynandra* indicated MFCs of ≥ 0.5 mg/ml for Microsporum canis and *Trichophyton mentagrophytes*, and ≥ 0.125 mg/ml for *Trichophyton rubrum* as result of subculturing 200 µl of dermatophytes from each serially diluted test tubes onto SDA slants. Mc = *Microsporum canis*; Tm = *Trichophyton mentagrophytes*; and, Tr = *Trichophyton rubrum*

**Table 3:** Percentage turbidity (mean) of aqueous and ethanol extracts against *M. canis, T. mentagrophytes*, and *T. rubrum*

	Aqueous extract			Ethanol extract		
Dilution (mg/ml)	Mc	Tm	Tr	Mc	Tm	Tr
0.125	50	50	0	50	25	0
0.0625	50	50	25	50	50	0
0.0313	75	50	50	100	50	0
0.0156	75	50	50	100	75	50
0.00781	100	100	100	100	100	100
0.00391	100	100	100	100	100	100

The lowest average percentage turbidity was exhibited by the aqueous and ethanol extracts against *Trichophyton rubrum*, followed by *Trichophyton mentagrophytes*, and finally *Microsporum canis*. Mc = Microsporum canis; Tm = *Trichophyton mentagrophytes*; and, Tr = *Trichophyton rubrum*. The cell entries (0-100) indicate the percentage turbidities (a measure of fungal growth of the three strains Mc, Tm and Tr), resulting from the effect of the two extracts at the corresponding dilutions. All tests were done in triplicate

The variable X1 = (Tr, Mc, and Tm) and dilution had a statistically significant effect on the outcome (turbidity) with associated *p*-values of 0.0083 and <0.0001, respectively. Extract (*X*2) = (aqueous extract and ethanol extract) was not found to be statistically significant on the outcome of turbidity (*p* = 0.8960). In addition, aqueous extract was associated 1.1 times (95 % CL 0.27 – 4.55) with a better outcome compared to ethanol extract although it was not statistically significant. Tr was more sensitive than Mc (*p* < 0.002) and more sensitive than Tm (*p* < 0.035) to the antifungal activity of *C. gynandra*. Tr was also 6.9 times (95 % CL: 1.15 – 41.6) more likely to have a better outcome (more sensitive) than Tm. Dilution had a strongly significant effect on the turbidity seen as an outcome (*p* < 0.0001). A summary of the analysis of maximum likelihood estimates obtained by fitting a proportional odds model is shown in Table 4.

**Table 4:** Mc, and Tm are compared to Tr and the aqueous extract is compared to ethanol extract when the model is fitted. Tr was more sensitive than Mc (p<0.002) and more sensitive than Tm (p<0.035) to the antifungal activity of *C. gynandra.* Comparing the aqueous extract and ethanol extract, there was no statistically significant difference in the effect of extract on fungal strains (p=0.8960). However, dilution had a strongly significant effect on the outcome (p<0.0001)

Parameter	Estimate	Standard Error	Wald Chi-Square	*P*-value
Mc	3.16	1.03	9.38	0.002*
Tm	1.93	0.92	4.47	0.035*
Aqueous extract	−0.10	0.72	0.02	0.896
Dilution	−57.11	14.32	15.90	<0.0001*

## Discussion

The present work was an initial screening study to verify the ethnomedicinal use of *Cleome gynandra* L., a plant used as an antifungal topical treatment for *Tinea capitis. Cleome gynandra* is a popular unconventional leafy vegetable [4, 5, 21] that largely grows wild and is used in Uganda to treat a variety of ailments including *Tinea capitis* [2, 3]. The phytochemical profile of *C. gynandra* in our study was similar to that of a recent study [22] that evaluated the antibacterial activity of *C. gynandra* and other plants. In another study, alkaloids, cyanidins, steroids, and reducing sugars were present in *Cleome gynandra* extracts [23] and these secondary metabolites were indicated to have antifungal activity. Additionally, phytochemical screening of dried powdered leaves of *Cleome gynandra* has shown the presence of flavonoids, saponins, tannins, sugars, cyanogenetic and cardiac glycosides, and the absence of anthroquinones and alkaloids in both ethanol and water plant extracts [24]. As indicated, the phytochemical analysis of *C. gynandra* shoot extracts in the present study demonstrates the presence of a diverse class of compounds known to have biological antifungal activities [25, 26].

Safety studies of the plant extracts were not carried out as mandated for clinical studies, but then the WHO guidelines, 1996 [27], state “*If a product has been traditionally used without demonstrated harm, no specific restrictive laboratory action should be undertaken unless new evidence demands a revised risk-benefit assessment.*” *C. gynandra* met this criterion, since it is a plant that traditionally is used both as a vegetable food and as a medicine.

At least one previous preliminary antimicrobial assay showed poor susceptibility of *Candida albicans* to inhibition or to the killing effect of a qualitative crude methanolic extract of *Cleome gynandra* [6]. The results obtained in our study, however, for the minimum inhibitory concentrations and for the minimum fungicidal concentrations, suggest that *C. gynandra* extracts possess antifungal activity. It is possible that the difference in activity described for the study that showed weak activity of *C. gynandra* extract and our study was because of the difference in the amount of active principles extracted, as well as the type of organism. MICs of both aqueous and ethanolic extracts ranged from 0.0313 to 0.5 mg/ml. There was a systematic trend of antifungal activity displayed by *C. gynandra* ethanol extract against the clinical fungal isolates. *T. rubrum* was most sensitive to both extracts followed by *T. mentagrophytes*, and least by *M. canis*. The water extract exhibited the lowest MFCs ranging from 0.0313 – 0.0625 mg/ml for *M. canis,* 0.0156 – 0.0313 mg/ml for *T. mentagrophytes*, and 0.0625 – 0.125 mg/ml for *T. rubrum*.

On the basis of the MICs obtained, the fungal isolates were generally sensitive to both the ethanol extract and the aqueous extract. Studies on other plants with antifungal activity have reported antifungal activities of alkaloids and flavonoids [23, 28], which were also present in the two extracts used in this study. The comparable antifungal activity of the water and ethanol extracts in this study suggests that the concentrations of the active metabolites extracted by the two solvents is not reduced by using water which yielded less extract than ethanol, and considering that the aqueous extract lacked oils, fats and steroids. This study has demonstrated that *Cleome gynandra* possesses antifungal activity and adds to the body of existing knowledge about this plant.

The present study was limited, however, in that it was an in vitro study, not an in vivo one. In addition, antifungal activity was evaluated on clinical isolates that already could have had prior exposure to conventional antifungal drugs. It is conceivable therefore, that *Cleome gynandra* extracts would have greater antifungal effect than was seen in this study. Lastly, in vitro testing to determine the end-points of MIC and MFC in the present study was for 14 days; however, in traditional medicinal use the plant leaves are applied topically as a remedy until the symptoms of *Tinea capitis* disappear, not necessarily for only 14 days. Future studies shall address the antifungal efficacy of *Cleome gynandra* extracts on *T. capitis* and other fungal conditions, through clinical trials comparing these extracts to standard antifungal agents.

## Conclusion

Both plant extracts demonstrated antifungal activity. Antifungal activity of the two plant extracts varied with the type of organism of the clinical fungal isolates. The ethanol extract showed comparable antifungal activity to the aqueous extract. There is need to develop new antifungal drugs that are cheap, accessible, safe and efficacious. Further quantitative studies should be done to determine the efficacy of specific active phytochemicals and exact concentrations of specified extracts per species of dermatophytes.

## Abbreviations

ATCC, American Type Culture Collection; CLSI, Clinical and Laboratory Standards Institute; Fgo, fungal growth; MFC, minimum fungicidal concentration; MIC, minimum inhibitory concentration; Nfgo, no fungal growth; RPMI, Roswell Park Memorial Institute; SDA, Sabouraud Dextrose Agar.
